# Mortality Event in Rainbow Snakes Linked to Snake Fungal Disease, United States

**DOI:** 10.3201/eid3111.250547

**Published:** 2025-11

**Authors:** Dane A. Conley, Gaëlle Blanvillain, Jaimie L. Miller, Kate E. Langwig, John D. Kleopfer, Jeffrey M. Lorch, Joseph R. Hoyt

**Affiliations:** Virginia Polytechnic Institute and State University, Blacksburg, Virginia, USA (D.A. Conley, G. Blanvillain, K.E. Langwig, J.R. Hoyt); University of Wisconsin–Madison, School of Veterinary Medicine, Madison, Wisconsin, USA (J.L. Miller); Virginia Department of Wildlife Resources, Henrico, Virginia, USA (J.D. Kleopfer); US Geological Survey, National Wildlife Health Center, Madison, Wisconsin, USA (J.M. Lorch)

**Keywords:** Rainbow snakes, fungi, snake fungal disease, fungal pathogen, mycosis, Ophidiomyces ophidiicola, Farancia erytrogramma, ophidiomycosis, United States

## Abstract

We report mortality in rainbow snakes in Virginia and North Carolina, USA, linked to snake fungal disease caused by *Ophidiomyces ophidiicola*. During 2013–2023, we observed 46 dead rainbow snakes with lesions indicative of snake fungal disease, noted elevated disease severity compared with other species, and recorded fewer live snakes over time.

Detecting and assessing declines in elusive or rare species can be difficult. Early identification of populations in decline can help accelerate intervention strategies and reduce the likelihood of genetic bottlenecks, population extirpation, and trophic disturbances of ecologically important species (*1*). Snake fungal disease (SFD) is caused by the fungal pathogen *Ophidiomyces ophidiicola* and affects a broad range of snake species (*2*), causing skin lesions as the fungus invades tissues, sometimes leading to impaired movement, anorexia, and even death (*3*). Researchers have documented population impacts from SFD in 2 snake species (*4*,*5*), but the extent of mortality across snake species is likely underestimated due to the cryptic nature of snakes. We describe a multiyear mortality event associated with SFD in a rare species, the rainbow snake (*Farancia erytrogramma*), in Virginia and North Carolina, USA.

In spring 2019, regional biologists from the Back Bay region of North Carolina and Virginia reported 6 deceased rainbow snakes. In spring of 2020, we found an additional 6 snakes in the same area (Figure 1, panel A). After those events, we gathered additional records of dead rainbow snakes (Appendix Table 1) from the region and began regular surveys and sampling of rainbow snakes and other species for *O. ophidiicola* in 2020–2023 (Appendix).

**Figure 1:**
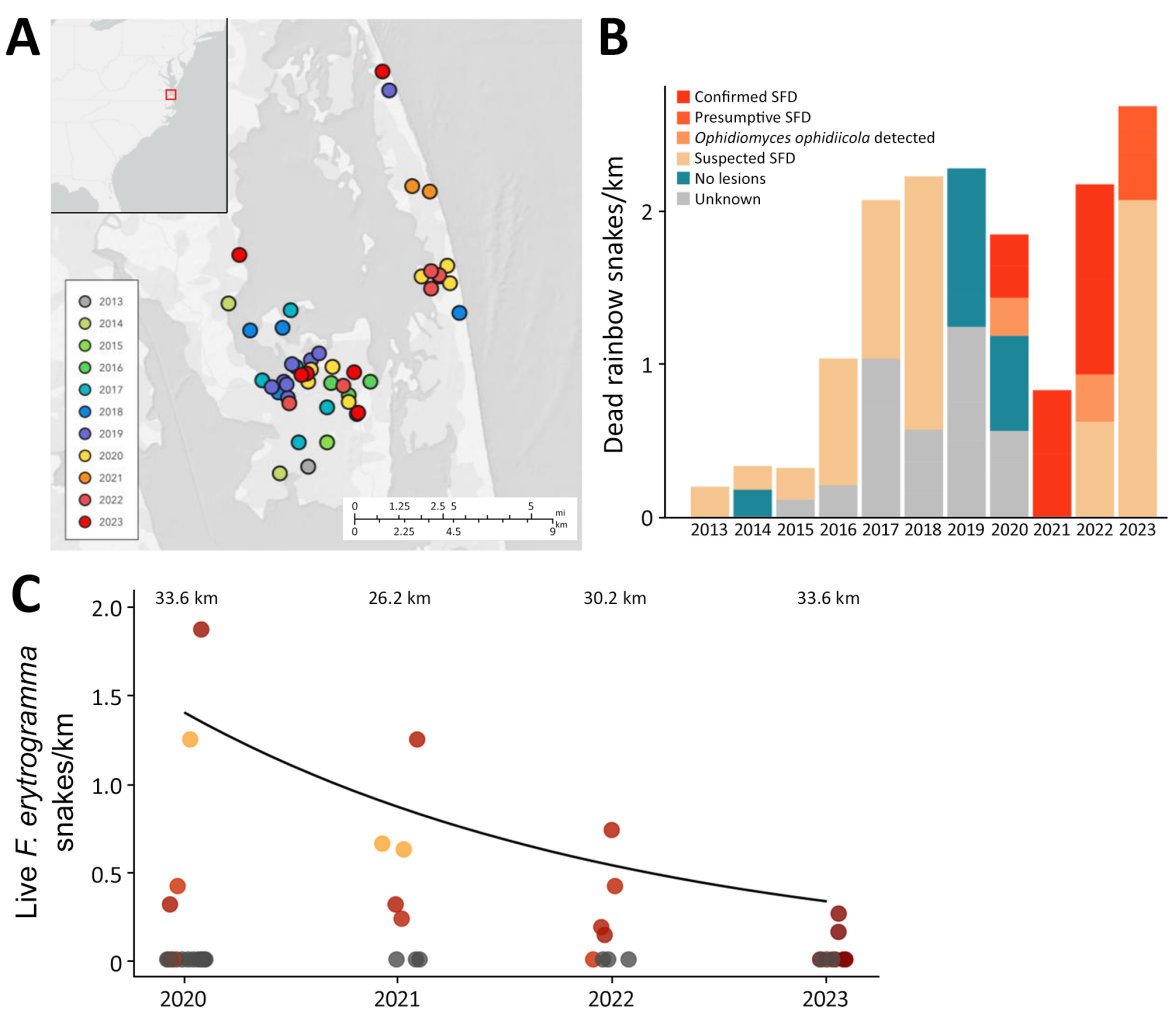
Encounters with dead and live snakes over time from a study of a mortality event in rainbow snakes linked to SFD, United States. A) Map of locations where dead *Farancia erytrogramma* rainbow snakes were observed during 2013*–*2023. Exact locations were jittered to obscure sensitive habitat. B) Stacked bar plot showing dead rainbow snakes found per kilometer in the Back Bay region of Virginia and North Carolina. Dead snakes characterized based on strength of evidence that they died from SFD: suspected, snakes with characteristic SFD lesions in photographs but no screening for *Ophidiomyces ophidiicola* performed; presumptive, snakes with characteristic SFD lesions, detection of *O. ophidiicola*, but no necropsies performed; confirmed, snakes with characteristic SFD lesions, detection of *O. ophidiicola*, and characteristic histologic lesions confirmed through histology; *O. ophidiicola* detected, snakes with no apparent lesions but tested positive for *O. ophidiicola;* no lesions, snakes with no apparent lesions in photographs and not tested for *O. ophidiicola*; unknown, snakes with no photographs of the dorsal and ventral sides. C) Number of live rainbow snakes encountered in the field per kilometer of transect surveyed with an incorporated 20-coverboard array during 2020–2023 (n = 19; zero-inflated Poisson log-scale year coefficient = −0.482 ± 0.226; p = 0.033) (Appendix Table 3). Total kilometers surveyed per year represented above survey data points. Color shading corresponds to mean infection severity (red is more severe than orange) during sampling event when the species was detected. Gray indicates sampling events without live rainbow snakes detected. SFD, snake fungal disease.

We captured and swabbed snakes in accordance with previously published protocols (*6*) and used sterile procedures for disinfecting equipment (Appendix). We extracted DNA from and tested samples using quantitative PCR (qPCR) to determine the presence of *O. ophidiicola*, according to established methods (*6*,*7*). To quantify lesion severity for all live-captured snakes, we used an approach integrating snake size, lesion size, and lesion progression (Appendix Table 2, Figure 1).

All dead rainbow snakes screened by qPCR (n = 9) were positive for *O. ophidiicola* and had skin lesions characteristic of SFD (Figure 1, panel B). Necropsies on a subsample (n = 6) indicated all snakes examined had lesions consistent with *O. ophidiicola* infection, including thickening of the epidermis by eosinophilic necrotic cellular debris containing fungal hyphae. Snakes also had invasion by hyphae in deeper tissue, including the dermis (6/6) (Appendix Figure 2, panel A), underlying skeletal muscle (3/6), and in oral and nasal epithelium and tooth pulp (1/6). We also observed hyphae or fibrin thrombi within blood vessels in the dermis (5/6). Most snakes (4/6) were in good body condition, had large amounts of fat, and showed no signs of other serious pathologic processes. We considered SFD as the ultimate cause of death in all 6 snakes. 

We also examined the number of live rainbow snakes captured over time using sampling data from standardized surveys conducted in 2020–2023 to assess preliminary trends while accounting for effort (Figure 1, panel C). We found a general decrease in the probability of detecting live rainbow snakes over time (log-scale year coefficient = −0.482 ± 0.226; p = 0.033) (Figure 1, panel C; Appendix Tables 3, 4).

A broader comparison of *O. ophidiicola* prevalence and severity of infection among other snakes in the community revealed that rainbow snakes were among the most infected species (Figure 2): *O. ophidiicola* prevalence was 80.1% (95% CIs 62.5%–92.5%), and log_10_ lesion severity was −1.71 (95% CI −1.94 to −1.50) (Appendix). Several other snake species also had notably high prevalence of *O. ophidiicola* and did not differ greatly from rainbow snakes (Figure 2, panel A) but were not found dead as part of the ongoing mortality event. Although live rainbow snakes were rare within the broader snake community (total live captures 16.4% [95% CI 11.3%–23%]; n = 25/153), they were disproportionately represented among dead snakes (87.5% [95% CI 52.9%–97.8%]; n = 7/8) found during the same period (2020–2023).

**Figure 2:**
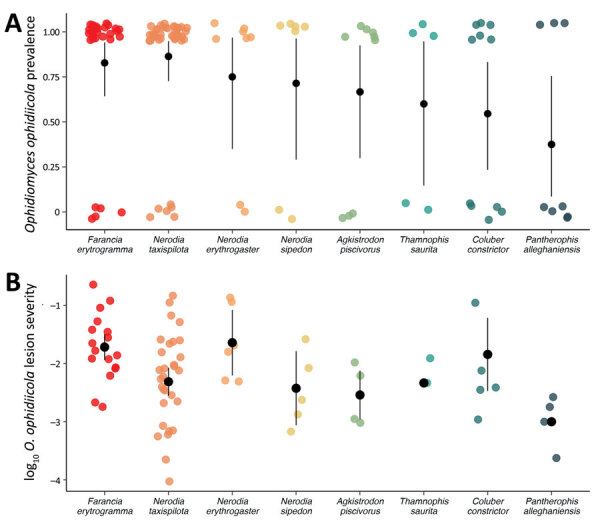
Variation in *Ophidiomyces ophidiicola* infection among snake species from a study of a mortality event in rainbow snakes linked to snake fungal disease, United States**.** Sampling results during spring (January−June) in 2020*–*2023 in the Back Bay watershed in Virginia and North Carolina. Black circles and error bars indicate mean lesion severity with 95% CIs. A) Each colored point represents an individual snake sampled and whether it was positive (1) or negative (0) for *O.* *ophidiicola*. Data points are slightly jittered for visualization purposes. B) Summed lesion severity values accounting for lesion size, lesion progression, and proportion of snake affected (Appendix). Snakes without lesions were omitted.

Observing wildlife mortality without obvious cause is rare and can indicate a more serious problem (*8*). We documented mortality of rainbow snakes using photographic, molecular, and histologic evidence, providing support that infection with *O. ophidiicola* is likely responsible. The rainbow snakes are considered a species of conservation concern, and although mortality in the species appeared to be ongoing as of 2023, the full extent of population declines remains uncertain.

*O. ophidiicola* was likely introduced to the United States in the early 1900s, although new (and possibly more virulent) strains have emerged recently (*9*). Increases in rainbow snake mortality could be the result of the introduction of more virulent strains of *O. ophidiicola* or shifts in environmental conditions since 2014 (*10*), but it is unclear why rainbow snakes appear particularly susceptible to infection (Figure 2). Nonetheless, the observed epizootic from a pathogen that has existed in North America for decades suggests SFD remains a threat to snake populations, which are a critical ecologic component of many ecosystems. Further research on the potential effects of *O. ophidiicola* would help clarify the impacts and trends of this disease on snake populations. Our study highlights the potential impact of disease-causing fungi such as *O. ophidiicola* on unmonitored, cryptic snake species like the rainbow snake.

Appendix Additional information for study of mortality event in rainbow snakes linked to snake fungal disease, United States.
