# Supplementary material for: Mortality Event in Rainbow Snakes Linked to Snake Fungal Disease, United States
Source: Emerg Infect Dis. 2025 Nov;31(11):2190–4. doi: 10.3201/eid3111.250547 (PMC12704546; doi:10.3201/eid3111.250547)
Supplement: Appendix — Additional information for study of mortality event in rainbow snakes linked to snake fungal disease, United States. [file 25-0547-Techapp-s1.pdf]

Article DOI: http://doi.org/10.3201/eid3111.250547

EID cannot ensure accessibility for supplementary materials supplied by authors. Readers who have difficulty accessing supplementary content should contact the authors for assistance.

Mortality Event in Rainbow Snakes Linked to Snake Fungal Disease, United States

Appendix

Materials and Methods

Population surveys and historical records

In addition to our own records (n = 21), we collected observations from regional biologists (n = 24), and iNaturalist (n = 1) of deceased *Farancia erytrogramma*. We then obtained the number of observers, effort (distance searched on foot in kilometers), location, whether snakes had visible skin lesions, and obtained photos of any snakes encountered when available using both our own data and from regional biologists that had initially submitted mortality reports. For the records of dead snakes recovered before 2018, distance was inconsistently recorded, however, the number of hours individuals searched for snakes was available for surveys. To estimate distance for surveys where only hours were available, we ran a linear regression model between total hours searched and survey distance for surveys between 2020 and 2023 when both metrics were consistently recorded (*coeff*: 0.447 ± 0.075, $p < 0.0001$, $R^2 = 0.27$). We then used the equation (distance (km) = intercept+slope*hours or distance = 1.362+0.447* hours) to predict the approximate distance for surveys where only hours had been recorded. For observations from regional biologists and iNaturalist, we used photos to both confirm species identity and determine whether animals had lesions typical of *Ophidiomyces ophidiicola* infection (Appendix Figure 2, panel B). Snakes with evident causes of death such as vehicle strikes were excluded from the mortality dataset due to poor condition of the animal for identifying lesions and swabbing. Diagnostic criteria for deceased *F. erytrogramma* (Figure 1, panel B) are based on previously established classification (*1*,*2*).

Ophidiomyces ophidiicola sampling methods

We collected skin swabs (n = 9) and performed necropsies (n = 6) on a subset of the 46 deceased *F. erytrogramma* collected from 2020–2023 to determine pathogen presence and the cause of mortality (Appendix Table 1). Beginning in 2020, we conducted targeted snake fungal disease (SFD) sampling of all snake species while focusing efforts on *F. erytrogramma* in the Back Bay region of Virginia and North Carolina. We captured snakes through visual encounters on walking standardized routes (n = 113/157) or driving transects (n = 5/157) and through coverboard arrays (n = 39/157), which consisted of one array of 20 (1.2 m x 2.2 m; 1.9 cm) plywood sheets placed in the sun adjacent to water. Multiple methods were used to minimize sampling bias and diversify the species sampled. Live *F. erytrogramma* were detected during walking standardized routes (n = 17/25) and under coverboards (n = 8/25). We observed no mortality beneath coverboards for any species. We conducted sampling in the spring during the peak infectious period (*3*). Upon capture, an epidermal swab dipped in 100 μL of sterile water, was run along the face, back and forth twice. The same swab was then run along the dorsal and ventral of the snake five times back and forth in a rotating motion. A separate "lesion swab" was run along any lesion present on the animal; this was used for confirmation screening for the presence of *Ophidiomyces ophidiicola.* Snakes that had either epidermal or lesion swab with *O. ophidiicola* were considered positive for the pathogen. Snout-vent-length (SVL) and tail-length (TL) were measured with a disinfected flexible fiberglass measuring tape. The circumference between the mid-body and snout (anterior circumference), mid-body (mid-body circumference), and mid-body and vent (posterior circumference) were recorded to calculate the surface area of the animal. Circumference was taken instead of diameter because many species flatten their bodies to appear larger as a defensive adaptation, which could result in less accurate measurements. Snakes that were greater than 20 cm snout-vent length (SVL) were implanted with 8 mm Biomark Passive Integrated Transponder (PIT) tags (Biomark, Boise, Idaho) so individuals could be differentiated if recaptured. Snakes that were recaptured in the same week were not sampled to avoid duplicated sampling and recaptures among weeks were very low (n = 4 / 153). Snakes were processed and released within 20 minutes of capture to minimize stress. Latex or nitrile gloves were worn to prevent cross contamination. For disinfection, we soaked non-electronic equipment in 10% bleach for a minimum of two minutes to ensure destruction of *O. ophidiicola*, and electronic equipment was disinfected using 70% ethanol (*4*).

Molecular testing

Using 250 µL Prepman Ultra Sample Preparation Reagent (Applied Biosystems, Foster City, California) with 10 mg of zirconium/silica beads, we extracted DNA from the collected swabs with cycles of 45 second homogenization, 30 second 13,000 RPM centrifuge, with a 10-minute 100°C heat bath. This was followed by a 2-minute cooling period and 3-minute 13,000 RPM centrifuge (*5*,*6*). To control for contamination, we prepared extraction negative controls using solely 250 µL of PrepMan Ultra Sample Preparation Reagent and 10 mg of zirconium/silica beads. We pipetted 50 µL to 100 µL of supernatant into a 96-well plate and preserved it in a −80°C freezer until a quantitative polymerase chain reaction (qPCR) assay could be run. Following (*5*), we performed real-time qPCR with Quantifast mastermix (Qiagen, Germantown, MD, USA) by amplifying a portion of the internal transcribed spacer 2 region of the ribosomal RNA gene specific for *O. ophidiicola* using 5 µL of the extracted DNA. To confirm the qPCR detection, we ran plates in duplicate to ensure accurate results. A positive in at least one of two plate replicates from an epidermal or lesion swab was considered a positive detection of *O. ophidiicola*. We generated a standard curve for each qPCR plate using six concentrations of IDT gBlock to produce standard curves of 500 fg to 0.005 fg of synthetic DNA in triplicate. Using Quantifast mastermix, the final reaction volume was 25 µL. Cycling conditions were: 95°C for 3 minutes, 95°C for 3 seconds, and 60°C for 30 seconds for a total of 40 cycles.

Quantification of infection severity

We assessed how infection severity varied among snake species to better understand how infections in *F. erytrogramma* compared to other snake species in the Back Bay area. To quantify severity of lesions for all live captured individuals, we used a scoring metric that incorporated both the surface area of the snake covered in lesions and the severity of each lesion (Appendix Table 2, Appendix Figure 1). We estimated the total surface area of each snake by measuring snout-vent length, tail length, and circumferences at three locations (anterior, mid-body, and posterior). We used the formula for the surface area of a cylinder to calculate surface area of each snake with the snout-vent length (SVL) of the individual ($radius = C/2\pi$); ($Area = 2\pi r2+2\pi rSVL$) with the addition of the tail surface area which was calculated using the posterior circumference and tail length (TL) with the surface area formula of a cone $Area = \pi r(r+ \sqrt{(TL^2+r^2)})$. We then measured individual lesion surface area for all lesions

occurring on each snake by directly measuring each lesion (*6*). We also scored lesion severity (*7*), giving each lesion a score from 1 to 5 (Appendix Table 2, Appendix Figure 1). We then multiplied severity scores (Appendix Table 2) with lesion surface area measurements for each lesion and summed these products to estimate the total severity of infection for each individual snake (e.g., y-axis in Figure 2, panel B). We $\log_{10}$ transformed the severity score values for graphs for easier visualization. Lesions were typically observed in the field, described qualitatively, photographed and scored from the photographs and descriptions. Similar scoring systems for *O. ophidiicola* have been previously developed, however these metrics do not account for lesion progression (*8*) or differences in body size (*2*).

Methodology of histology

Tissues for histologic evaluation were fixed in 10% neutral buffered formalin, decalcified in a saturated ethylenediaminetetraacetic acid (EDTA) solution (i.e., head containing skull), trimmed, embedded in paraffin, and sectioned at 5 microns. The hematoxylin and eosin method were used to stain internal organs, whereas skin samples were stained with the periodic acid-Schiff method.

Statistical analysis

We modeled the encounter rate of live *F. erytrogramma* using a zero-inflated Poisson model with a log link function. The response variable was the number of live snakes detected per survey, and the predictor was year to assess annual trends. To account for variation in survey effort across transects, we used the natural log of transect length as an offset, standardizing the response to the number of live *F. erytrogramma* per km searched. We used a zero inflated Poisson because model checks indicated a Poisson only model underfit 0s (ratio = 0.93), and the zero-inflated Poisson was not overdispersed (dispersion ratio = 1.4, $p = 0.26$). As an additional check, we also examined the probability a *F. erytrogramma* was found alive or dead (1|0) using a binomial model with year as a predictor and the natural log of transect length as an offset. Both models were fit using the glmmTMB package in RStudio.

We calculated prevalence estimates among species and 95% confidence intervals using the binom.test() function in R (Appendix Figure 2, panel A). We also examined differences in prevalence among species using a generalized linear model with a binomial distribution and a logit link with the probability of infection (1|0) as our response variable and species as a

predictor. We calculated average lesion severity and derived 95% confidence intervals by adding or subtracting 1.96 times the standard error from the mean lesion severity (Figure 2, panel B). We made statistical comparisons of severity among snakes using a linear regression of $\log_{10}$ transformed total lesions severity scores.

Results

Species comparisons

For prevalence of *Ophidiomyces ophidiicola (Oo)*, most other snake species did not differ significantly from *F. erytrogramma*, however *P. alleghaniensis* did have a significantly lower prevalence (*coeff:* −2.08 ± 0.88, p = 0.018). Four species had significantly lower severity scores compared to *F. erytrogramma: Agkistrodon piscivorus* (*coeff:* −0.823 ± 0.375, p = 0.031), *Nerodia sipedon* (*coeff:* −0.748 ± 0.343, p = 0.032), *N. taxispilota* (*coeff:* −0.550 ± 0.202, p = 0.008), and *Pantherophis alleghaniensis* (*coeff:* −1.270 ± 0.375, p = 0.001). *Nerodia erythrogaster* had slightly higher severity, but this difference was not significant (*coeff:* 0.069 ± 0.320; p = 0.831).

Any use of trade, firm, or product names is for descriptive purposes only and does not imply endorsement by the U.S. Government.

References

1. Davy CM, Shirose L, Campbell D, Dillon R, McKenzie C, Nemeth N, et al. Revisiting ophidiomycosis (snake fungal disease) after a decade of targeted research. Front Vet Sci. 2021;8:665805. PubMed

2. Baker SJ, Haynes E, Gramhofer M, Stanford K, Bailey S, Christman M, et al. Case definition and diagnostic testing for snake fungal disease. Herpetol Rev. 2019;50:279–85.

3. McKenzie JM, Price SJ, Fleckenstein JL, Drayer AN, Connette GM, Bohuski E, et al. Field diagnostics and seasonality of *Ophidiomyces ophiodiicola* in wild snake populations. Ecohealth. 2019;16:141–50. PubMed

4. Rzadkowska M, Allender MC, O'Dell M, Maddox C. Evaluation of Common Disinfectants Effective against *Ophidiomyces ophiodiicola*, the Causative Agent of Snake Fungal *Disease.* J Wildl Dis. 2016;52:759–62. PubMed

5. Bohuski E, Lorch JM, Griffin KM, Blehert DS. TaqMan real-time polymerase chain reaction for detection of *Ophidiomyces ophiodiicola*, the fungus associated with snake fungal disease. BMC Vet Res. 2015;11:95. PubMed

6. Blanvillain G, Lorch JM, Joudrier N, Bury S, Cuenot T, Franzen M, et al. Contribution of host species and pathogen clade to snake fungal disease hotspots in Europe. Commun Biol. 2024;7:440. PubMed

7. Lorch JM, Lankton J, Werner K, Falendysz EA, McCurley K, Blehert DS. Experimental infection of snakes with *Ophidiomyces ophiodiicola* causes pathological changes that typify snake fungal disease. mBio. 2015;6:e01534–15. PubMed

8. McCoy CM, Lind CM, Farrell TM. Environmental and physiological correlates of the severity of clinical signs of snake fungal disease in a population of pigmy rattlesnakes, *Sistrurus miliarius.* Conserv Physiol. 2017;5:cow077. PubMed

Appendix Table 1. Summary of deceased *Farancia erytrogramma* (n = 46) including initial observation dates and categorization of *Ophidiomyces ophidiicola (Oo)* presence, as referenced in Figure 1, panel A. SFD = snake fungal disease.

Farancia erytrogramma mortality observations					
**Date**	**Species**	**Category**	**Date**	**Species**	**Category**
5/18/13	*Farancia erytrogramma*	Suspected SFD	4/12/19	*Farancia erytrogramma*	Suspected SFD
4/13/14	*F. erytrogramma*	Suspected SFD	10/23/19	*F. erytrogramma*	No Lesions
4/13/14	*F. erytrogramma*	No Lesions	10/28/19	*F. erytrogramma*	No Lesions
2/9/15	*F. erytrogramma*	Suspected SFD	3/4/20	*F. erytrogramma*	No Lesions
4/9/15	*F. erytrogramma*	Unknown	4/11/20	*F. erytrogramma*	Unknown
3/15/16	*F. erytrogramma*	Suspected SFD	4/11/20	*F. erytrogramma*	Unknown
3/25/16	*F. erytrogramma*	Suspected SFD	4/29/20	*F. erytrogramma*	Confirmed SFD
7/21/16	*F. erytrogramma*	Unknown	4/29/20	*F. erytrogramma*	Confirmed SFD
2/12/17	*F. erytrogramma*	Unknown	5/12/20	*F. erytrogramma*	Oo Detected
2/20/17	*F. erytrogramma*	Unknown	12/4/20	*F. erytrogramma*	Unknown
3/28/17	*F. erytrogramma*	Suspected SFD	3/12/21	*F. erytrogramma*	Confirmed SFD
4/1/17	*F. erytrogramma*	Unknown	5/6/21	*F. erytrogramma*	Confirmed SFD
5/15/17	*F. erytrogramma*	Suspected SFD	3/21/22	*F. erytrogramma*	Oo Detected
3/30/18	*F. erytrogramma*	Suspected SFD	3/28/22	*F. erytrogramma*	Suspected SFD
4/26/18	*F. erytrogramma*	Unknown	3/28/22	*F. erytrogramma*	Suspected SFD
5/10/18	*F. erytrogramma*	Unknown	4/11/22	*F. erytrogramma*	Confirmed SFD
5/10/18	*F. erytrogramma*	Unknown	4/11/22	*F. erytrogramma*	Confirmed SFD
6/1/18	*F. erytrogramma*	Suspected SFD	3/1/23	*F. erytrogramma*	Presumptive SFD
3/24/19	*F. erytrogramma*	Unknown	3/4/23	*F. erytrogramma*	Presumptive SFD
3/24/19	*F. erytrogramma*	Unknown	3/4/23	*F. erytrogramma*	Presumptive SFD
3/24/19	*F. erytrogramma*	Unknown	4/5/23	*F. erytrogramma*	Suspected SFD
3/24/19	*F. erytrogramma*	Unknown	4/18/23	*F. erytrogramma*	Suspected SFD
3/24/19	*F. erytrogramma*	Unknown	5/4/23	*F. erytrogramma*	Suspected SFD

Appendix Table 2. Lesion scoring 1–5 for snakes sampled with corresponding description of individual lesions.

Individual lesion severity score	Individual lesion description
1	Disrupted/mild distortion of scales and/or mild edema of individual scales
2	Mild crusting/thickening on portions of individual scales (covering <33% of skin surface within an affected area) or small nodules (suspected granulomas) in the skin
3	Moderate crusting of scales (covering 33%–75% of skin surface within an affected area). The epidermal layer of the skin is still attached with no exposure of living layers of skin.
4	Crusts cover the majority of infected scales (>75% - 95% of the area); sloughing of affected tissue resulting in exposure of living layers of skin or muscle tissue.
5	Crusts encompassing multiple scales such that individual scales within the affected area of skin are no longer recognizable, sloughing of affected tissue resulting in exposure of living layers of skin or muscle tissue, and/or crust covering eye or occluding nostril or loreal pit.

Appendix Table 3. Statistical output from zero-inflated Poisson model of live rainbow snakes over time with a standardized transect and coverboards.

**Zero-inflated Poisson -** Formula: faer_live ~ year + offset(log(km_hiked)); Zero inflation: ~1				
**Parameter**	**Estimate**	**Std. error**	**z-value**	**Pr(>|z|)**
Intercept	974.6541	456.5484	2.135	0.0328
Year	−0.4828	0.2259	−2.138	0.0325
**Zero-inflation model**				
Intercept	−0.7258	0.8673	−0.837	0.403

Appendix Table 4. Statistical output from binomial model of the probability of encountering a live rainbow snake over time using a standardized transect with coverboards.

**Binomial logistic regression** - Formula: presence ~ year + offset(log(km_hiked))				
**Parameter**	**Estimate**	**Std. error**	**z-value**	**Pr(>|z|)**
Intercept	3549.2634	1319.8907	2.689	0.00717
Year	−1.7554	0.6527	−2.689	0.00716

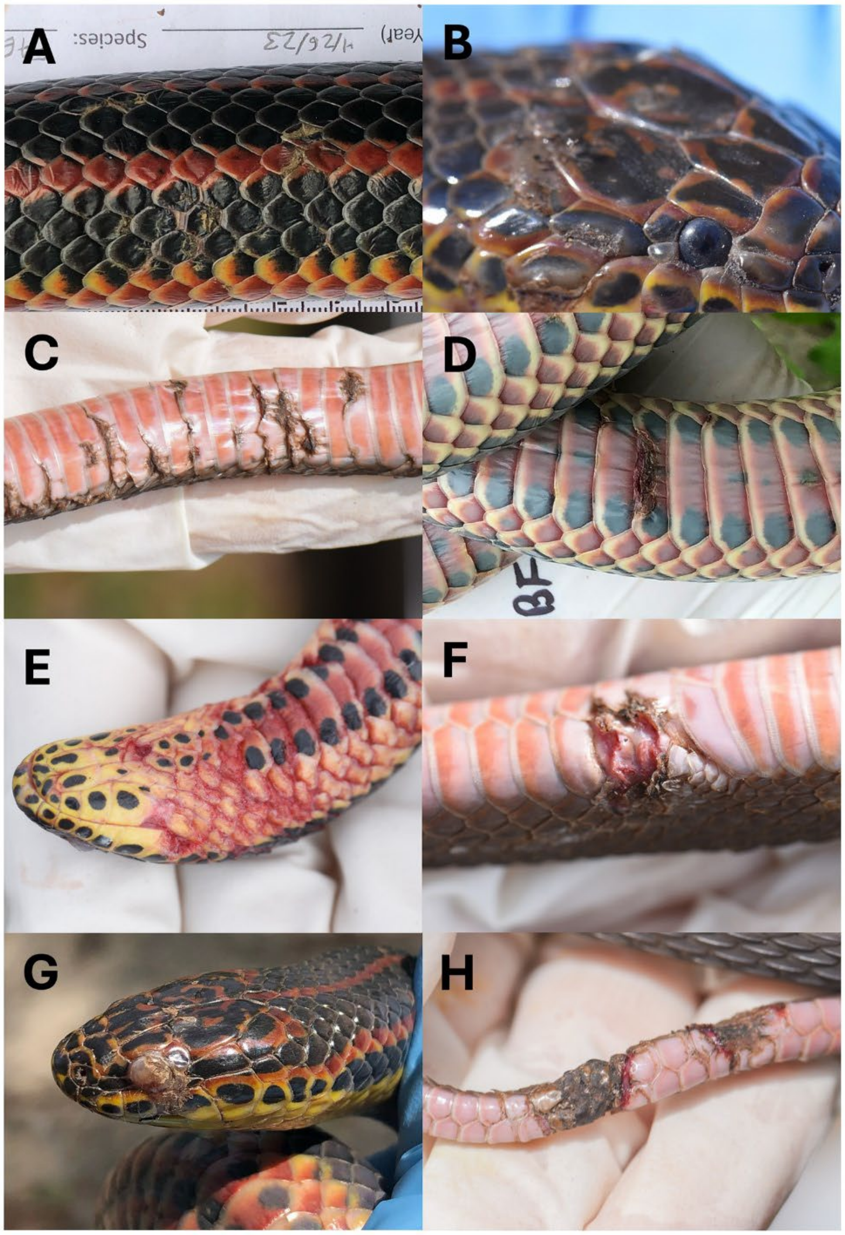

Appendix Figure 1. Examples of lesions with corresponding severity score. (A) Mildly disfigured scales on a rainbow snake, *Farancia erytrogramma,* (severity score: 1). **(**B) Mild crusting on the face of *F. erytrogramma*, with epidermis intact (severity score: 2). (C) Crust covering most of the affected area of skin on a plain-bellied watersnake, *Nerodia erythrogaster* (severity score: 3). (D) Crust covering most of the affected area of skin on a *F. erytrogramma* (severity score: 3). (E) Small exposed layers of living skin on the ventral of the neck of a *F. erytrogramma* (severity score: 4). (F) Exposed live tissue likely resulting from detachment of crusted skin near the vent of a *N. erythrogaster* (severity score: 4). (G) Left eye of a *F. erytrogramma* that is covered with thickened skin, rendered the snake blind (severity score: 5). (H) Tail of a *N. erythrogaster* with crust encompassing multiple scales and exposing living layers of skin (severity score: 5).

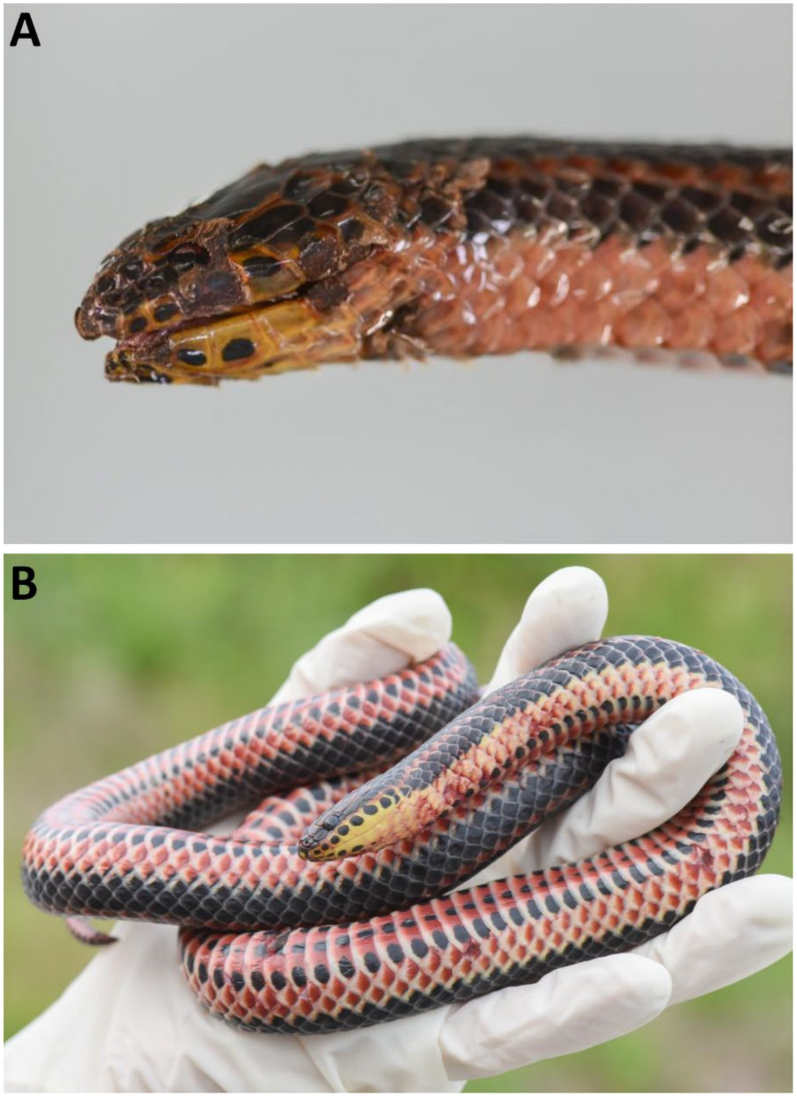

Appendix Figure 2. *Farancia erytrogramma* from Back Bay, Virginia. (A) One of the 6 necropsied *F. erytrogramma,* showing severe lesions caused by *Ophidiomyces ophidiicola* on the head. (B) A live *F. erytrogramma* that was found during active during the day that exhibited multiple lesions across its body, with a notable concentration on the ventral region.
